# *CHRNA7* Polymorphisms and Dementia Risk: Interactions with Apolipoprotein ε4 and Cigarette Smoking

**DOI:** 10.1038/srep27231

**Published:** 2016-06-02

**Authors:** Pei-Hsuan Weng, Jen-Hau Chen, Ta-Fu Chen, Yu Sun, Li-Li Wen, Ping-Keung Yip, Yi-Min Chu, Yen-Ching Chen

**Affiliations:** 1 Department of Family Medicine, Taiwan Adventist Hospital, Taipei, Taiwan; 2 Institute of Epidemiology and Preventive Medicine, College of Public Health, National Taiwan University, Taipei, Taiwan; 3 Department of Geriatrics and Gerontology, National Taiwan University Hospital, Taipei, Taiwan; 4 Department of Neurology, National Taiwan University Hospital, Taipei, Taiwan; 5 Department of Neurology, En Chu Kong Hospital, New Taipei City, Taiwan; 6 Department of Laboratory Medicine, En Chu Kong Hospital, New Taipei City, Taiwan; 7 School of Medicine, Fu-Jen Catholic University, New Taipei City, Taiwan; 8 Center of Neurological Medicine, Cardinal Tien Hospital, New Taipei City, Taiwan; 9 Department of Laboratory Medicine, Cardinal Tien Hospital, New Taipei City, Taiwan; 10 Department of Public Health, College of Public Health, National Taiwan University, Taipei, Taiwan; 11 Research Center for Genes, Environment and Human Health, College of Public Health, National Taiwan University, Taipei, Taiwan

## Abstract

α7 nicotinic acetylcholine receptor (α7nAChR, encoded by *CHRNA7*) is involved in dementia pathogenesis through cholinergic neurotransmission, neuroprotection and interactions with amyloid-β. Smoking promotes atherosclerosis and increases dementia risk, but nicotine exerts neuroprotective effect via α7nAChR in preclinical studies. No studies explored the gene-gene, gene-environment interactions between *CHRNA7* polymorphism, apolipoprotein E (*APOE*) ε4 status and smoking on dementia risk. This case-control study recruited 254 late-onset Alzheimer’s disease (LOAD) and 115 vascular dementia (VaD) cases (age ≥65) from the neurology clinics of three teaching hospitals in Taiwan during 2007–2010. Controls (N = 435) were recruited from health checkup programs and volunteers during the same period. Nine *CHRNA7* haplotype-tagging single nucleotide polymorphisms representative for Taiwanese were genotyped. Among *APOE* ε4 non-carriers*, CHRNA7* rs7179008 variant carriers had significantly decreased LOAD risk after correction for multiple tests (GG + AG vs. AA: adjusted odds ratio = 0.29, 95% confidence interval = 0.13–0.64, *P* = 0.002). Similar findings were observed for carriers of GT haplotype in *CHRNA7* block4. A significant interaction was found between rs7179008, GT haplotype in block4 and *APOE* ε4 on LOAD risk. rs7179008 variant also reduced the detrimental effect of smoking on LOAD risk. No significant association was found between *CHRNA7* and VaD. These findings help to understand dementia pathogenesis.

The growing number of dementia patients has introduced a tremendous healthcare burden in the aging society. Alzheimer’s disease (AD) is the most common type of dementia, followed by vascular dementia (VaD). AD pathology is characterized by the selective loss of nicotinic acetylcholine receptors (nAChR)^1^ and elevated amyloid-β (Aβ) deposition in hippocampus and neocortex^2^. Reduced nAChR was also reported in the subcortical regions of VaD^3^.

α7nAChR is one of the most important nAChR subunits in the central nervous system and is often co-localized with Aβ deposition in the neuritic plaques of AD cortical neurons^4^. α7nAChR plays a pivotal role in dementia development through enhancing cholinergic neurotransmission^5^, inducing long-term potentiation^6^ and exerting neuroprotective effect^7^. However, as dementia progresses, elevated Aβ binds to α7nAChR with high affinity, inactivating α7nAChR and inhibiting its neuroprotective effect^8^. Another well-known risk factor of dementia is the cigarette smoking habit^9^, which increases dementia risk probably through accelerating atherosclerosis^10^. In contrast, the nicotine compound has a beneficial effect on cognition as it exerts neuroprotective action via α7nAChR in preclinical studies^11^. Taken together, complex interactions should exist among α7nAChR, Aβ, and cigarette smoking in the pathogenesis of dementia, which are currently unknown.

α7nAChR is encoded by *CHRNA7* gene on chromosome 15q13–14, which is a region linked to several neuropsychiatric disorders, including bipolar affective disorder, schizophrenia, parkinsonism, several types of epilepsy, and autism^12^. *CHRNA7* polymorphisms were also associated with decreased AD risk^13^ and slower progression from mild cognitive impairment to AD^14^, but other studies reported non-significant findings^15^,^16^,^17^,^18^,^19^,^20^. The inconsistence across studies may be attributable to differences in ethnicity and lack of information on gene–gene or gene–environment interactions. Apolipoprotein E *(APOE)* ε4, an essential genetic risk factor of late-onset AD (LOAD), is associated with increased Aβ deposition^21^. Despite prior *in vitro* evidence suggesting an important interaction between Aβ peptide and α7nAChR in the pathogenesis of dementia^8^, no studies explored the interaction between *APOE* ε4 and *CHRNA7* polymorphisms on AD risk. Meanwhile, nicotine is an agonist of α7nAChR, but no studies examined whether the association of *CHRNA7* polymorphisms with dementia varied depending on the smoking status.

This case-control study examined the association between *CHRNA7* polymorphisms and dementia risk. Nine haplotype-tagging single nucleotide polymorphisms (htSNP) representative for Taiwanese were genotyped, capturing a majority of genetic information of *CHRNA7*. Interactions among *CHRNA7* genotypes, *APOE* ε4, and dementia (LOAD, VaD) were explored. Stratified analyses were further performed by smoking history.

## Materials and Methods

### Study participants

Dementia patients were recruited from the neurology clinics of three teaching hospitals in northern Taiwan (National Taiwan University Hospital, En Chu Kong Hospital, and Cardinal Tien Hospital) from November 2007 to July 2010. Healthy controls were recruited from geriatric health checkup programs and from volunteers during the same period of time. All participants were Taiwanese (Han Chinese descents) who were 65 years and older. The exclusion criteria were participants with a history of depression, Parkinson’s disease, stroke, brain tumor, lack of blood sample, or poor DNA quality. After exclusion, a total of 254 LOAD cases, 115 small-vessel VaD cases, and 435 controls were included in the statistical analyses. All of the study protocols were approved by the Institutional Review Boards of National Taiwan University Hospital (200709031R, 200712102R), En Chu Kong Hospital (ECKIRB:98015), and Cardinal Tien Hospital (CTH-96-2-030). Informed consents were obtained from all subjects. Written consents were obtained from participants who were able to give consent by themselves, and from legal guardian/next of kin for those who couldn’t give consent themselves due to severe cognitive impairment. All of the experiments were carried out in accordance with the guidelines of the World Medical Association Declaration of Helsinki.

A detailed questionnaire was administered to all participants via a face-to-face interview with the assistance of informants. The collected information included data on demography, lifestyle, and comorbidity. Detailed smoking history (starting age of smoking habit, years of smoking, and years since quitting smoking) was obtained from the questionnaire. Ever-smokers were defined as those having smoked ≥100 cigarettes during their lifetime. Previous studies found good reliability between self-reported smoking status and elevated nicotine-related biomarkers in the body^22^. Blood samples were collected in EDTA tubes and genomic DNA was extracted from the buffy coat by using the QuickGene-Mini80 kit (Fujifilm, Tokyo, Japan) after centrifugation.

### Dementia Evaluation

Probable LOAD was diagnosed by experienced neurologists as per the criteria defined by the National Institute of Neurological and Communicative Disorders and Stroke and the Alzheimer’s Disease and Related Disorders Association^23^. Brain images (computed tomography or magnetic resonance imaging) were performed to exclude organic brain lesions. VaD was diagnosed using the criteria of the National Institute of Neurological Disorders and Stroke-Association Internationale pour la Recherche et l’Enseignement en Neurosciences^24^. Because of different etiology between large- and small-vessel VaD, only patients with small-vessel VaD (e.g., lacunar infarction and leukoaraiosis) were included in this study in order to provide a more homogeneous outcome.

Mini-Mental State Examination (MMSE) was used to evaluate the cognitive performance of LOAD and VaD cases^25^. The controls were assessed by Short Portable Mental Status Questionnaire (SPMSQ) with the objective of excluding participants with possible cognitive impairment^26^. To further ensure that the controls were cognitively intact, only those without memory complaints and completely independent in performing activities of daily living and instrumental activities of daily living were included.

### SNP Selection and Genotyping Assays

Common (frequency ≥5%) SNPs in *CHRNA7* were selected from Han Chinese in Beijing (CHB) genotype data from the International HapMap Project (http://hapmap.ncbi.nlm.nih.gov/). Haplotype blocks were determined by the Haploview program (http://www.broadinstitute.org/haploview/haploview) using a modified Gabriel algorithm^27^,^28^. htSNPs were selected from each haplotype block using the tagSNP program with R^2^ > 0.7 in each haplotype block^29^. Genotypes of *CHRNA7* SNPs were determined by TaqMan® Genomic Assays using the ABI 7900HT fast real-time PCR system (Applied Biosystems Inc., Foster City, CA, USA). *APOE* genotypes were determined by the assay developed by Chapman *et al.*^30^. The *APOE* diplotypes (ε2/ε2, ε2/ε3, ε3/ε3, ε2/ε4, ε3/ε4, and ε4/ε4) were determined by *APOE*112 (rs429358) and *APOE*158 (rs7412)^31^. *APOE* ε4 carriers were defined by participants carrying ε2/ε4, ε3/ε4, or ε4/ε4 diplotypes. Participants carrying other diplotypes (ε2/ε2, ε2/ε3, and ε3/ε3) were defined as *APOE* ε4 non-carriers. The genotyping call rate was greater than 95% for each SNP. The internal genotyping quality control obtained from 5% of samples in duplicates had a concordance rate of 100%.

### Statistical Analyses

The Student’s t test (for normally-distributed continuous variables), Mann-Whitney U test (for non-normally distributed continuous variables), and χ^2^ test (for categorical variables) were used to compare the distribution of potential confounders by LOAD, VaD, and controls. The Hardy–Weinberg equilibrium (HWE) test in controls was performed for each SNP of *CHRNA7* and *APOE* genes to examine possible genotyping errors or selection bias. The expectation-maximization algorithm was applied to estimate haplotype frequencies^29^. Participants were stratified by intervals of 5-years of age and cases were compared with controls within each age stratum in the multivariable analysis. Age (in years) was further adjusted in the multivariable analysis to control for residual confounding within each age stratum. Conditional logistic regression models were used to estimate the adjusted odds ratio (AOR) and 95% confidence interval (CI) for dementia (LOAD or VaD) in participants carrying 1 or 2 versus 0 copies of the minor allele of each SNP and each multilocus haplotype after adjustment for age, sex, *APOE* ε4, and education year.

Because *APOE* ε4 is an important risk factor for dementia and due to the *in vitro* evidence of interactions between Aβ and α7nAChR^8^, stratification analysis was performed by *APOE* ε4 status (carriers vs. non-carriers). The type I error resulting from multiple tests was controlled by false discovery rate (FDR)^32^.

To compare the joint effects of *CHRNA7* polymorphisms and the smoking status on LOAD, four categories were created for each SNP and haplotype (non-variant carriers who ever smoked/never smoked and variant carriers who ever smoked/never smoked). Non-variant carriers who never smoked served as the reference group.

Because the numbers of homozygous variants were small and most of the included SNPs followed dominant mode of inheritance, all analyses were performed under dominant models. All statistical analyses were performed using SAS 9.4 (SAS Institute, Cary, NC, USA). A two-sided *P* < 0.05 was considered to be statistically significant.

## Results

### Population Characteristics

This study included 254 LOAD patients, 115 small-vessel VaD patients, and 435 healthy controls. Compared with controls separately, LOAD and VaD patients were significantly older (79.8 and 79.8 vs. 73.2 years-old, respectively), less educated (≥6 years of education: 49% and 40% vs. 88%, respectively), and more likely to be ever-smokers (24% and 28% vs. 17%, respectively). Compared with controls, LOAD patients showed a higher number of females (64% vs. 53%, respectively) and *APOE* ε4 carriers (39% vs. 15%, respectively), less hypertension rate (38% vs. 53%, respectively) and hypercholesterolemia (18% vs. 31%, respectively). VaD patients had more hypertension percentage (65% vs. 53%) and diabetes mellitus (35% vs. 14%) compared with the controls (Table 1).

### Haplotype-tagging SNPs in *CHRNA7* gene

**Figure 1:**
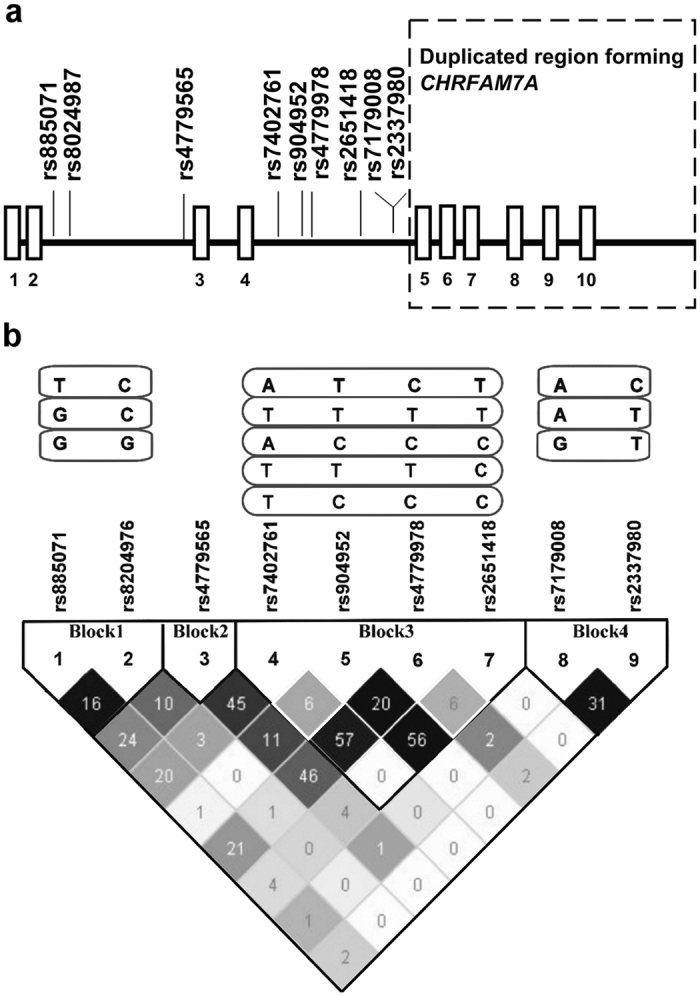
*CHRNA7* genomic structure and linkage disequilibrium (LD) plot. (**a**) shows the genomic structure of the *CHRNA7* gene and the htSNPs in this study. The dashed rectangle indicates the region duplicated in the fusion gene, *CHRFAM7A*. Exons and introns are indicated by boxes and lines, respectively (not to scale). (**b**) shows the LD plot generated by Haploview program using genotype data from this study. Levels of pairwise D′, which indicate the degree of LD between two htSNPs, are shown in the LD structure in gray scale. Levels of pairwise r^2^, which indicate the degree of correlation between two SNPs, are shown as the number in each cell. Common (frequency ≥5%) haplotypes were identified in each haplotype block. A modified Gabriel *et al.* algorithm was used to define the haplotype block.

Nine common (frequency ≥5%) htSNPs, forming four haplotype blocks in the *CHRNA7* gene, were selected and genotyped [Table 2, Fig. 1(a)]. Block1 contained two htSNPs (SNP1: rs885071, SNP2: rs8024987), block2 contained one htSNP (SNP3: rs4779565), block3 contained 4 htSNPs (SNP4: rs7402761, SNP5: rs904952, SNP6: rs4779978, SNP7: rs2651418), and block4 contained 2 htSNPs (SNP8: rs7179008, SNP9: rs2337980). The linkage disequilibrium (LD) structure is shown in Fig. 1(b). The minor allele frequencies (MAFs) for the nine htSNPs among controls ranged from 0.09 to 0.44 (Table 2), which were similar to those of the MAFs of Han Chinese from HapMap database (http://hapmap.ncbi.nlm.nih.gov/) and can reflect the genetic distribution among general Chinese population. None of the *CHRNA7* SNPs were out of HWE after correction for multiple tests.

### Association between *CHRNA7* htSNPs or haplotypes and Dementia

The association between *CHRNA7* polymorphisms and dementia was examined after adjustment for age, sex, *APOE* ε4 status, and education year. Three common (frequency ≥5% among controls) haplotypes were identified in *CHRNA7* haplotype block1. Block2 included only one htSNP and was consequently excluded from the haplotype analysis. Five common haplotypes were identified in block3. Block4 included three common haplotypes. The effects of *CHRNA7* SNPs and haplotypes in block4 on LOAD risk are shown in Table 3. Rs7179008 [htSNP in block4 (SNP8)] and GT haplotype in block4 [consisting of rs7179008 (SNP8) and rs2337980 (SNP9)] were associated with decreased LOAD risk (AOR = 0.50, 95%, CI = 0.28–0.92, *P* = 0.02; AOR = 0.49, 95% CI = 0.27–0.90, *P* = 0.02, Table 3). The effects of haplotypes in block1 and block3 on LOAD risk are shown in Supplementary Table S1. A protective effect against LOAD risk was also found among carriers of TC haplotype in block1 (AOR = 0.51, 95% CI = 0.30–0.86, Supplementary Table S1).

The effects of *CHRNA7* SNPs and haplotypes on VaD risk are shown in Table 4 and Supplementary Table S2 separately. Rs904952 (SNP5) was associated with increased VaD risk (AOR = 1.77, 95% CI = 1.01–3.09, *P* = 0.046, Table 4). TTTC haplotype in block3 was associated with decreased VaD risk (AOR = 0.33, 95% CI = 0.12–0.88, Supplementary Table S2). However, none of the above SNPs or haplotypes was significantly associated with LOAD or VaD after correction for multiple tests using FDR.

### Modification Effect by *APOE* ε4 Status

A significant interaction was found between the variant rs7179008 (SNP8) and *APOE* ε4 on LOAD risk (*P*_interaction _= 0.03, Table 3). The effect of GT haplotype in block4 on LOAD risk was also significantly modified by *APOE* ε4 status (*P*_interaction_ = 0.03, Table 3). Among *APOE* ε4 non-carriers, carriers of variant rs7179008 (AOR = 0.29, 95% CI = 0.13–0.64, *P* = 0.002 Table 3) and GT haplotype in block4 (AOR = 0.28, 95% CI = 0.13–0.63, *P* = 0.002, Table 3) demonstrated decreased LOAD risk compared with non-carriers. This decreased risk remained significant after correction for multiple tests using FDR based on 18 independent tests (9 SNPs by *APOE* ε4 status). No significant associations were observed between any of the *CHRNA7* htSNPs, haplotypes, and LOAD among *APOE* ε4 carriers (Table 3).

The carriers of variant rs904952 (SNP5) were associated with increased VaD risk among *APOE* ε4 non-carriers (AOR = 2.26, 95% CI = 1.18–4.33, *P* = 0.01, Table 4)), but the result became non-significant after correction for multiple tests.

### Modification Effect by Smoking

No significant interaction was observed between rs7179008 (SNP8) variant and smoking on LOAD risk (*P*_interaction_ = 0.70). However, significant associations were found in a few subgroups. Ever-smokers had significantly increased LOAD risk after adjustment for age, sex, *APOE* ε4 status, and education year (AOR = 1.89, 95% CI = 1.01–3.52, *P* = 0.045). Among participants carrying rs7179008 wild-type, ever-smokers had increased LOAD risk compared with never-smokers (AOR = 2.00, 95% CI = 1.03–3.87, *P* = 0.008, Table 5). However, the detrimental effect of smoking was diminished if ever-smokers carried variant rs7179008 (AOR = 0.79, 95% CI = 0.21–2.96, *P* = 0.70, Table 5) compared with non-smokers with wild-type rs7179008.

## Discussion

To the best of our knowledge, this is the first study exploring the gene–gene and gene–environment interactions of *CHRNA7* polymorphisms on dementia risk. We found that the variants of *CHRNA7* rs7179008 (SNP8) and GT haplotype in block4 significantly protect against LOAD among *APOE* ε4 non-carriers. The effects of rs7179008 and GT haplotype in block4 on LOAD risk were significantly modified by *APOE* ε4 status. The variant rs7179008 decreased the detrimental effect of smoking on LOAD risk. No significant associations were observed between *CHRNA7* polymorphisms and VaD.

The analysis of *CHRNA7* genome is complex due to its interaction with the highly polymorphic *CHRFAM7A* gene^33^. During evolution, exons 5–10 of *CHRNA7* were duplicated and formed the fusion gene, *CHRFAM7A*^33^,^34^. The subunits encoded by *CHRFAM7A* lack part of the ligand binding site, and *CHRFAM7A* works as a dominant negative regulator on α7nAChR ion channel function^35^. Rs7179008 is located at intron 4 of *CHRNA7* gene, which is a common breakpoint for gene rearrangement and may thus contribute to the polymorphisms of *CHRFAM7A*^34^,^35^. The variations in *CHRFAM7A* may in turn affect the expression of α7nAChR. Besides affecting α7nAChR expression by pre-mRNA alternative splicing^36^, it is also possible that this intronic SNP acts through affecting subsequent protein production^36^, or through LD with other functional genetic regions. GT haplotype in block4 consisted of rs7179008 and rs2337980, which explains the protective effect of this haplotype.

An Irish study previously found that TCC haplotype in *CHRNA7* block1 was significantly associated with reduced AD risk^13^, which consisted of rs1514246, rs2337506, and rs8027814. Another European study found −86 C/T promoter polymorphism in *CHRNA7* gene was associated with slower progression from mild cognitive impairment to AD^14^. The genome-wide association studies by Heinzen *et al.*^16^ and Swaminathan *et al.*^15^ found that the variants of *CHRNA7* seem to contribute to AD risk and warrant further investigation. But the other candidate gene association studies^19^,^20^ and genome-wide association studies^17^,^18^ yielded non-significant results regarding the effect of *CHRNA7* polymorphisms on dementia risk. These inconsistent findings may be related to different SNPs in Caucasians^13^,^15^,^16^,^17^,^18^,^19^ or lack of information on gene–gene and gene–environment interactions. Important genetic variants might be missed under gene-environment interactions, when the genetic association is opposite among different subgroups^37^. Because *APOE* ε4 and smoking are both well-established common risk factors for dementia and the abundant *in vitro* evidence suggesting their interactions with α7nAChR, our study takes the interactions into account. In addition, our study was adjusted for important risk factors for dementia (age, sex, *APOE* ε4, and education year), which was not considered in most of the previous studies^13^,^16^,^19^,^20^.

The following mechanisms may explain how *CHRNA7* polymorphisms protect against LOAD risk through affecting the expression of α7nAChR. α7nAChR forms ligand-gated ion channels on neuron cell membranes, which are activated by the neurotransmitter acetylcholine or other agonists, e.g., nicotine^5^. When ligands bind to α7nAChR ion channel, the influx of sodium depolarizes cell membranes and increases cholinergic neurotransmission^5^. Presynaptic α7nAChR also modulates the release of other neurotransmitters^38^. Long-term potentiation is facilitated via α7nAChR^6^, which is important for memory consolidation. Furthermore, the stimulation of postsynaptic α7nAChR increases calcium influx and activates the intracellular signal transduction pathway, conferring neuroprotective effect by protecting neurons against Aβ toxicity^7^. In addition to disease prediction, our previous work has found that *CHRNA7* polymorphisms may predict cognitive response to cholinesterase inhibitors and serve as a pharmacogenomic marker in LOAD treatment^39^.

**Figure 2:**
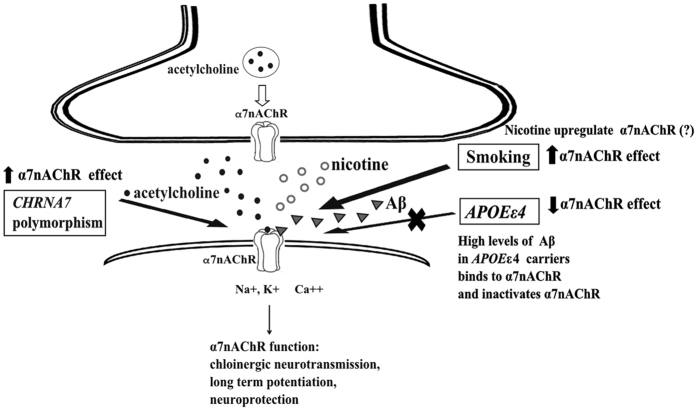
Postulated mechanism for the interaction between *CHRNA7* polymorphisms, *APOE* ε4 and smoking. *CHRNA7* encodes α7nAChR and may affect the pathogenesis of LOAD through the following mechanisms: (1) modulation of neurotransmitter release in presynaptic neurons^38^, (2) memory enhancement via mediating cholinergic neurotransmission^5^ and long-term potentiation^6^, and (3) neuroprotection via α7nAChR^7^. In *APOE* ε4 carriers, high levels of Aβ bind to α7nAChR, inactivating the receptor and decreasing its neuroprotective effect^8^. The nicotine in cigarette smoking is an agonist to α7nAChR which potentiates the neuroprotective effect of the receptor in preclinical studies^11^. Upregulation of α7nAChR was reported among smokers^44^. Abbreviations: nAChR, nicotinic acetylcholine receptor; *APOE*, apolipoprotein E; Aβ, amyloid β.

The postulated mechanisms explaining the interactions among *CHRNA7* polymorphisms, Aβ, and smoking on LOAD risk are shown in Fig. 2. *In vitro* evidence suggested an important interaction of Aβ and nAChR on AD pathogenesis^8^. As Aβ level increases pathologically with dementia progression, Aβ binds to α7nAChR with high affinity, which inactivates α7nAChR and decreases its neuroprotective effect^8^. Chen *et al.* also found Aβ impaired long term potentiation as a consequence of dysfunctional α7nAChR^40^. In addition, another *in vitro* study revealed that other *APOE*-derived peptides disrupt acetylcholine-mediated peak current response through the direct blockade of α7nAChR^41^. Compared with *APOE* ε4 non-carriers, *APOE* ε4 carriers showed increased Aβ deposits in brains^21^ and decreased nAChR binding sites^42^, which may further diminish the protection of α7nAChR. These facts corroborate our finding that the protective effect of the variant *CHRNA7* rs7179008 was only observed among *APOE* ε4 non-carriers.

The joint effect of smoking and *CHRNA7* polymorphisms on LOAD risk had not been explored previously. We found that the detrimental effect of smoking was attenuated among carriers of variant *CHRNA7* rs7179008. Accumulating evidence based on large prospective cohort studies revealed that smoking increases AD risk^9^, which was consistent with our findings. The increased risk may result from the pro-atherogenic effect of smoking that contributes to dementia progression^10^. α4β2nAChR, another common nAChR subunit in the central nervous system, binds to nicotine with high affinity. Many studies have demonstrated increased numbers of high-affinity nAChRs in the brains of smokers^43^,^44^. α7nAChR binds to nicotine with lower affinity. Despite preclinical studies found that nicotine enhances α7nAChR-mediated neuroprotection^45^, studies were inconsistent regarding whether the expression of α7nAChR is increased in the brains of smokers^46^. In a post-mortem brain biopsy, Mousavi *et al.* found a significantly increased α7nAChR protein levels in the temporal cortex of smokers compared with non-smokers^44^. Thus, nicotine might offset the harm of toxic compounds in cigarette smoke by increasing the protective effect of α7nAChR among *CHRNA7* variant carriers. Future studies are required to determine the level of nicotine that stimulates α7nAChR, the duration of persisted receptor upregulation after smoking cessation, and the possible modifying effect by disease status and genetic variation.

However, we found that *CHRNA7* polymorphisms were not associated with VaD risk, which was not previously reported as far as we know. Few studies examined the change of nAChRs during VaD pathogenesis. One study reported decreased α4β2nAChR expression in subcortical region of VaD patients^3^, but another study did not find the association^47^. Genetic susceptibility to VaD has been much less understood compared with AD. One possible cause is that different types of ischemic stroke and VaD (e.g., small-vessel occlusion and large artery atherosclerosis) are related to different genetic factors^48^,^49^. Thus, genetic analyses by VaD subtypes provide clearer explanation to different etiologies^50^. VaD subtypes were often neglected in previous genetic association studies. Therefore, in this study, we included only small-vessel VaD cases to minimize the heterogeneity.

This study presents a few strengths. To the best of our knowledge, for the first time, we explored the joint effects of *CHRNA7* polymorphisms, *APOE* ε4 status, and smoking on LOAD and VaD risk. Exploring the gene–gene and gene–environment interactions may help us further understand the pathogenesis of dementia. *CHRNA7* polymorphisms may be important genetic risk factors for *APOE* ε4 non-carriers. Second, in this study, we used a systematic approach to select htSNPs representative of Taiwanese, which captured abundant genetic information of *CHRNA7* gene (R^2^ > 0.7) and were different from SNPs selected for Caucasians. Finally, this study was adjusted for many important confounders, which makes our findings less biased.

This work also demonstrated some limitations. It included only 115 small-vessel VaD cases and may be underpowered to detect the genetic effect on VaD. Similarly, the joint effect of *CHRNA7* polymorphisms and smoking on LOAD risk needs to be interpreted with caution and should be regarded as exploratory and hypotheses generating, due to a relatively small number of smokers.

In summary, the variants of *CHRNA7* rs7179008 and GT haplotype in block4 were associated with reduced LOAD risk among *APOE* ε4 non-carriers. The association between *CHRNA7* polymorphisms and LOAD was substantially modified by *APOE* ε4 and smoking status. Future large studies are warranted to confirm our findings.

## Additional Information

**How to cite this article**: Weng, P.-H. *et al. CHRNA7* Polymorphisms and Dementia Risk: Interactions with Apolipoprotein ε4 and Cigarette Smoking. *Sci. Rep.* **6**, 27231; doi: 10.1038/srep27231 (2016).

## Supplementary Material

Supplementary Information

## Figures and Tables

**Table 1:** Characteristics of the study population.

		LOAD (N = 254)	VaD (N = 115 )	Control (N = 435)
		mean ± SD		
Age		**79.8 ± 6.3**	**79.8 ± 6.1**	73.2 ± 5.8
MMSE score		18.7 ± 5.1	15.2 ± 6.3	NA
SPMSQ (number of errors)		NA	NA	0.1 ± 0.4
		N (%)		
Female		**162 (64)**	66 (57)	232 (53)
Education	≦6 years	**128 (51)**	**69 (60)**	50 (12)
	6–12 years	**86 (34)**	**34 (30)**	171 (39)
	>12 years	**37 (15)**	**12 (10)**	212 (49)
Ever smoking		**60 (24)**	**32 (28)**	75 (17)
Alcohol consumption		28 (11)	19 (17)	48 (11)
Hypertension		**96 (38)**	**75 (65)**	232 (53)
Diabetes mellitus		48 (19)	**40 (35)**	62 (14)
Hypercholesterolemia		**46 (18)**	25 (22)	133 (31)
*APOE* ε4 status		**99 (39)**	26 (23)	65 (15)

Cases (LOAD or VaD) were compared to controls. Numbers in bold indicate significant findings (*P *< 0.05). Abbreviations: LOAD, late-onset Alzheimer’s disease; VaD, vascular dementia; SD, standard deviation; MMSE, Mini-Mental State Exmination; SPMSQ, Short Portable Mental Status Questionnaire; NA, not applicable; *APOE*, apolipoprotein E.

**Table 2:** Characteristics of *CHRNA7* haplotype-tagging SNPs.

Haplotype block	SNP name	rs no.	Nucleotide change	HapMap		Control		LOAD		VaD	
				CHB MAF	CEU MAF	MAF	HWE *p*	MAF	HWE *p*	MAF	HWE *p*
1	SNP1	rs885071	T→G	0.38	0.81	0.41	0.26	0.46	0.50	0.38	0.54
1	SNP2	rs8024987	C→G	0.11	0.24	0.11	0.05	0.15	0.14	0.11	0.79
2	SNP3	rs4779565	G→T	0.38	0.41	0.34	0.11	0.39	0.69	0.35	0.71
3	SNP4	rs7402761	A→T	0.45	0	0.44	0.37	0.44	0.79	0.44	0.32
3	SNP5	rs904952	T→C	0.29	0.53	0.27	0.16	0.34	0.16	0.33	0.13
3	SNP6	rs4779978	C→T	0.38	0.31	0.36	0.83	0.35	0.65	0.32	0.50
3	SNP7	rs2651418	T→C	0.42	0.53	0.39	0.62	0.42	0.41	0.44	0.89
4	SNP8	rs7179008	A→G	0.13	0.27	0.09	0.33	0.07	0.08	0.12	0.21
4	SNP9	rs2337980	C→T	0.26	0.50	0.24	0.03	0.25	0.69	0.23	0.96

All SNPs are intronic SNPs. Abbreviations: SNP, single nucleotide polymorphism; CHB, Han Chinese in Beijing, China;

CEU, Utah residents with ancestry from northern and western European; MAF, minor allele frequency; HWE, Hardy–Weinberg equilibrium test;

LOAD, late-onset Alzheimer’s disease; VaD, vascular dementia.

**Table 3:** Association between *CHRNA7* SNPs and LOAD by *APOE* ε4 status.

Haplotype block	SNP/Haplotype (frequency among controls)		0 copies		1 or 2 copies		*P*_*interaction*_
			Case/Control	AOR	Case/Control	AOR (95% CI)	
1	SNP1	All	76/145	1.00	178/290	1.48 (0.94–2.32)	0.23
		*APOE* ε4 (−)	44/121	1.00	110/247	1.25 (0.73–2.13)	
		*APOE* ε4 (+)	31/24	1.00	68/41	2.10 (0.89–4.94)	
1	SNP2	All	182/348	1.00	68/84	1.53 (0.93–2.52)	0.69
		*APOE* ε4 (−)	111/294	1.00	40/71	1.52 (0.84–2.75)	
		*APOE* ε4 (+)	70/52	1.00	28/13	1.68 (0.63–4.42)	
2	SNP3	All	95/179	1.00	157/255	1.16 (0.76–1.79)	0.62
		*APOE* ε4 (−)	59/155	1.00	94/212	1.26 (0.76–2.09)	
		*APOE* ε4 (+)	35/23	1.00	63/42	0.84 (0.35–2.02)	
3	SNP4	All	77/139	1.00	176/293	1.50 (0.73–1.82)	0.59
		*APOE* ε4 (−)	48/125	1.00	105/241	1.22 (0.72–2.09)	
		*APOE* ε4 (+)	28/14	1.00	71/50	0.84 (0.32–2.21)	
3	SNP5	All	115/225	1.00	134/205	1.34 (0.88–2.04)	0.15
		*APOE* ε4 (−)	64/189	1.00	51/35	1.59 (0.97–2.61)	
		*APOE* ε4 (+)	87/175	1.00	46/29	0.79 (0.35–1.81)	
3	SNP6	All	109/181	1.00	143/253	0.79 (0.51–1.21)	0.82
		*APOE* ε4 (−)	75/161	1.00	79/207	0.79 (0.48–1.29)	
		*APOE* ε4 (+)	34/20	1.00	63/44	0.65 (0.26–1.62)	
3	SNP7	All	88/160	1.00	165/274	1.10 (0.72–1.71)	0.23
		*APOE* ε4 (−)	48/135	1.00	105/232	1.30 (0.77–2.20)	
		*APOE* ε4 (+)	40/24	1.00	59/41	0.73 (0.32–1.66)	
4	SNP8	All	219/352	1.00	32/78	**0.50 (0.28–0.92)**	**0.03**
		*APOE* ε4 (−)	135/298	1.00	16/66	0.29(0.13–0.64)*	
		*APOE* ε4 (+)	83/52	1.00	16/12	1.32 (0.45–3.87)	
4	SNP9	All	143/260	1.00	110/174	1.07 (0.70–1.64)	0.10
		*APOE* ε4 (−)	93/221	1.00	60/146	0.87 (0.53–1.44)	
		*APOE* ε4 (+)	50/39	1.00	49/26	1.79 (0.78–4.07)	
4	Hap1: AC	All	14/33	1.00	240/402	1.37 (0.59–3.23)	0.61
	(76%)	*APOE* ε4 (−)	7/27	1.00	147/341	1.57 (0.56–4.41)	
		*APOE* ε4 (+)	7/6	1.00	92/59	0.99 (0.19–5.04)	
4	Hap2: AT	All	171/319	1.00	83/116	1.34 (0.84–2.12)	0.66
	(15%)	*APOE* ε4 (−)	109/271	1.00	45/97	1.28 (0.74–2.21)	
		*APOE* ε4 (+)	62/47	1.00	37/18	1.52 (0.62–3.73)	
4	Hap3: GT	All	222/357	1.00	32/78	**0.49 (0.27–0.90)**	**0.03**
	(9%)	*APOE* ε4 (−)	138/302	1.00	16/66	0.28 (0.13–0.63)*	
		*APOE* ε4 (+)	83/53	1.00	16/12	1.30 (0.44–3.83)	

All models were adjusted for age, sex, *APOE ε4*, and education year and conditional on 5-year age strata. Numbers in bold indicate significant findings (*P* < 0.05). *The association remained significant after correction for multiple tests by false discovery rate (FDR). The effects of *CHRNA7* haplotypes in block1 and block3 on LOAD risk are shown in Supplementary Table S1 because the results were non-significant after correction for multiple tests. Block2 included only one htSNP and was excluded from the haplotype analysis. Abbreviations: AOR, adjusted odds ratio; CI, confidence interval; SNP, single nucleotide polymorphism; *APOE*, apolipoprotein E; Hap, haplotype.

**Table 4:**
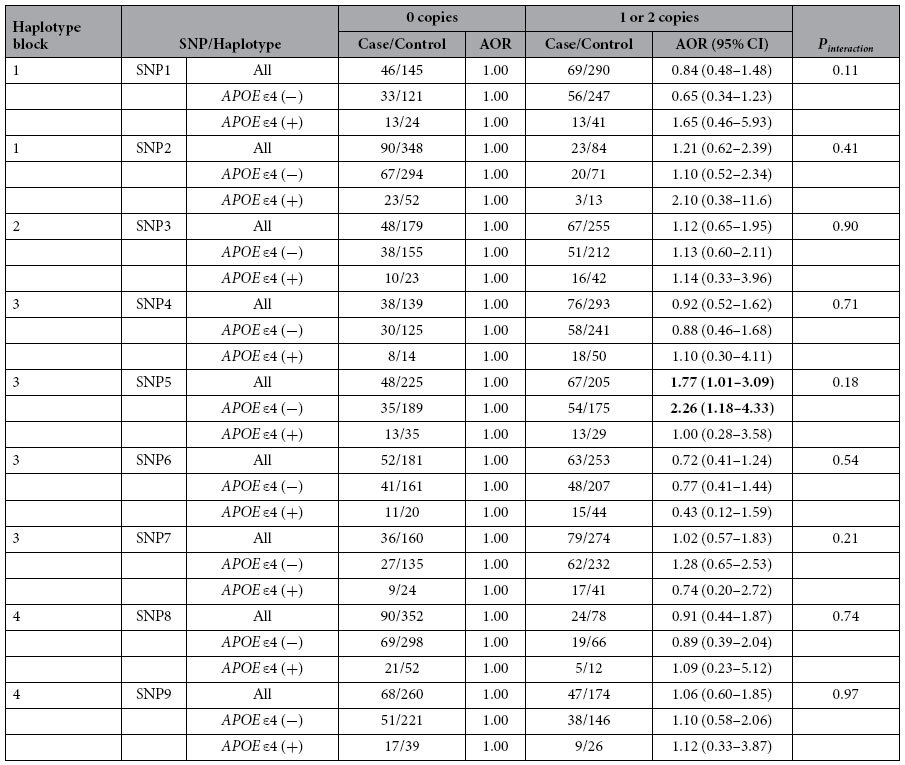
Association between *CHRNA7* SNPs and VaD by *APOE* ε4 status.

All models were adjusted for age, sex, *APOE* ε4, and education year and conditional on 5-year age strata. Numbers in bold indicate significant findings (*P* < 0.05). The results became non-significant after correction for multiple tests. Abbreviations: VaD, vascular dementia; AOR, adjusted odds ratio; CI, confidence interval; SNP, single nucleotide polymorphism; *APOE*, apolipoprotein E.

**Table 5:** Polymorphisms of *CHRNA7* rs7179008 and LOAD risk by smoking status.

	rs7179008 (SNP8)				*P*_interaction_
	0 copies (AA)		1 or 2 copies (AG+GG)		
	Case/Control	AOR (95% CI)	Case/Control	AOR (95% CI)	
Never-smokers	164/291	1.00	27/63	0.54 (0.27–1.04)	0.70
Ever-smokers	55/60	**2.00 (1.03–3.87)**	5/15	0.79 (0.21–2.96)	

All models were adjusted for age, sex, apolipoprotein E ε4, and education year and conditional on 5-year age strata. Numbers in bold indicate significant findings (*P* < 0.05). Abbreviations: AD, Alzheimer’s disease; SNP, single nucleotide polymorphism; AOR, adjusted odds ratio; CI, confidence interval.
