# Supplementary material for: CHRNA7 Polymorphisms and Dementia Risk: Interactions with Apolipoprotein ε4 and Cigarette Smoking
Source: Sci Rep. 2016 Jun 2;6:27231. doi: 10.1038/srep27231 (PMC4890170; doi:10.1038/srep27231)
Supplement: Supplementary Information [file srep27231-s1.pdf]

Supplementary information

CHRNA7 Polymorphisms and Dementia Risk: Interactions with Apolipoprotein ε4 and Cigarette Smoking

Pei-Hsuan Weng[1,2], Jen-Hau Chen[2,3], Ta-Fu Chen[4], Yu Sun[5], Li-Li Wen[6], Ping-Keung Yip[7,8], Yi-Min Chu[9] & Yen-Ching Chen[2,10,11*]

1 Department of Family Medicine, Taiwan Adventist Hospital, Taipei, Taiwan.
2 Institute of Epidemiology and Preventive Medicine, College of Public Health, National Taiwan University, Taipei, Taiwan.
3 Department of Geriatrics and Gerontology, National Taiwan University, Taipei, Taiwan.
4 Department of Neurology, National Taiwan University Hospital, Taipei, Taiwan.
5 Department of Neurology, En Chu Kong Hospital, Taipei, Taiwan.
6 Department of Laboratory Medicine, En Chu Kong Hospital, Taipei, Taiwan.
7 School of Medicine, Fu-Jen Catholic University, New Taipei City, Taiwan.
8 Center of Neurological Medicine, Cardinal Tien Hospital, Taipei, Taiwan.
9 Department of Laboratory Medicine, Cardinal Tien Hospital, Taipei, Taiwan.
10 Department of Public Health, College of Public Health, National Taiwan University, Taipei, Taiwan.
11 Research Center for Genes, Environment and Human Health, College of Public Health, National Taiwan University, Taipei, Taiwan.

*Correspondence to:
Yen-Ching Chen, ScD.
Institute of Epidemiology and Preventive Medicine,
National Taiwan University,
No.17 Xu-Zhou Road, Taipei 10055, Taiwan.
(O) 886-2-3366-8019
(Fax) 886-2-2351-1955
E-mail: karenchen@ntu.edu.tw

Supplementary Table S1. Association between *CHRNA7* haplotypes and LOAD by *APOE* ε4 status.

Haplotype Block	Haplotype (frequency among controls)		Dominant model				
			0 copies		1 or 2 copies		
			Case/ Control	AOR	Case/ Control	AOR (95% CI)	$P_{interaction}$
1	Hap1: TC	All	57/69	1.00	197/366	**0.51 (0.30-0.86)**	0.50
	(59%)	*APOE*ε4 (-)	35/58	1.00	119/310	**0.44 (0.24-0.82)**	
		*APOE*ε4 (+)	22/10	1.00	77/55	0.76 (0.28-2.07)	
1	Hap2: GC	All	121/209	1.00	133/226	1.47 (0.96-2.23)	0.33
	(30%)	*APOE*ε4 (-)	70/173	1.00	84/195	1.27 (0.77-2.08)	
		*APOE*ε4 (+)	49/36	1.00	50/29	1.88 (0.83-4.26)	
1	Hap3: GG	All	185/350	1.00	69/85	1.52 (0.92-2.51)	0.67
	(11%)	*APOE*ε4 (-)	114/296	1.00	40/72	1.50 (0.83-2.72)	
		*APOE*ε4 (+)	71/52	1.00	28/13	1.68 (0.64-4.47)	
3	Hap1: ATCT	All	137/206	1.00	117/229	0.70 (0.45-1.09)	0.28
	(31%)	*APOE*ε4 (-)	88/171	1.00	66/197	0.62 (0.37-1.04)	
		*APOE*ε4 (+)	48/34	1.00	51/31	1.19 (0.51-2.80)	
3	Hap2: TTTT	All	135/233	1.00	119/202	0.97 (0.63-1.50)	0.96
	(27%)	*APOE*ε4 (-)	87/202	1.00	67/166	0.96 (0.58-1.60)	
		*APOE*ε4 (+)	47/31	1.00	52/34	0.96 (0.42-2.23)	
3	Hap3: ACCC	All	146/274	1.00	108/161	1.22 (0.78-1.90)	0.17
	(20%)	*APOE*ε4 (-)	83/228	1.00	71/140	1.43 (0.86-2.39)	
		*APOE*ε4 (+)	62/44	1.00	37/21	0.75 (0.30-1.86)	
3	Hap4: TTTC	All	227/372	1.00	27/63	0.54 (0.28-1.05)	0.88
	(7%)	*APOE*ε4 (-)	139/318	1.00	15/50	0.57 (0.25-1.29)	
		*APOE*ε4 (+)	87/52	1.00	12/13	0.47 (0.16-1.44)	
3	Hap5: TCCC	All	219/382	1.00	35/53	1.40 (0.74-2.67)	0.86
	(6%)	*APOE*ε4 (-)	130/326	1.00	24/42	1.42 (0.67-2.98)	
		*APOE*ε4 (+)	87/55	1.00	12/10	1.17 (0.31-4.46)	

Numbers in bold indicate significant findings (*P* <0.05). The effects of *CHRNA7* haplotypes in block4 on LOAD risk are shown in Table 3 because the results remained significant after correction for multiple tests. Block2 included only one htSNP and was excluded from the haplotype analysis. Abbreviations: LOAD, late-onset Alzheimer's disease; AOR, adjusted odds ratio; CI, confidence interval; *APOE*, apolipoprotein E. Block 2 included only one haplotype-tagging single nucleotide polymorphism and thus was not included in the haplotype analysis. All models were adjusted for age, sex, *APOE* ε4, and education years and conditional on 5-year age strata.

Supplementary Table S2. Association between *CHRNA7* haplotypes and VaD by *APOE* ε4 status.

Haplo-type Block	Haplotype (frequency among controls)		Dominant model				
			0 copies		1 or 2 copies		
			Case/ Control	AOR	Case/ Control	AOR (95% CI)	$P_{interaction}$
1	Hap1: TC	All	18/60	1.00	97/365	1.05 (0.49-2.26)	0.21
	(59%)	*APOE*ε4 (-)	17/50	1.00	72/310	0.86 (0.37-2.00)	
		*APOE*ε4(+)	1/10	1.00	25/55	4.18 (0.40-44.0)	
1	Hap2: GC	All	60/208	1.00	55/225	0.88 (0.51-1.52)	0.50
	(30%)	*APOE*ε4 (-)	44/172	1.00	45/196	0.77 (0.41-1.43)	
		*APOE*ε4(+)	16/36	1.00	10/29	1.05 (0.29-3.83)	
1	Hap3: GG	All	92/349	1.00	23/84	1.19 (0.60-2.35)	0.39
	(11%)	*APOE*ε4 (-)	69/297	1.00	20/71	1.07 (0.50-2.28)	
		*APOE*ε4(+)	23/52	1.00	3/13	2.10 (0.38-11.7)	
3	Hap1: ATCT	All	63/205	1.00	52/228	0.86 (0.49-1.50)	0.94
	(31%)	*APOE*ε4 (-)	49/171	1.00	40/197	0.87 (0.46-1.65)	
		*APOE*ε4(+)	14/34	1.00	12/31	0.93 (0.28-3.03)	
3	Hap2: TTTT	All	63/234	1.00	52/199	0.97 (0.56-1.71)	0.89
	(27%)	*APOE*ε4 (-)	49/202	1.00	40/166	0.97 (0.51.1.85)	
		*APOE*ε4(+)	14/32	1.00	12/33	1.11 (0.30-4.05)	
3	Hap3: ACCC	All	66/274	1.00	49/159	1.34 (0.76-2.38)	0.72
	(20%)	*APOE*ε4 (-)	51/230	1.00	38/138	1.40 (0.73-2.69)	
		*APOE*ε4(+)	15/44	1.00	11/21	1.36 (0.36-5.06)	
3	Hap4: TTTC	All	103/373	1.00	12/60	**0.33 (0.12-0.88)**	0.42
	(7%)	*APOE*ε4 (-)	80/321	1.00	9/47	0.40 (0.13-1.20)	
		*APOE*ε4(+)	23/52	1.00	3/13	0.17 (0.02-1.48)	
3	Hap5: TCCC	All	95/381	1.00	20/52	1.63 (0.73-3.61)	0.61
	(6%)	*APOE*ε4 (-)	73/326	1.00	16/42	1.86 (0.76-4.59)	
		*APOE*ε4(+)	22/55	1.00	4/10	1.01 (0.13-7.91)	
4	Hap1: AC	All	6/33	1.00	109/400	1.30 (0.42-3.97)	0.86
	(76%)	*APOE*ε4 (-)	6/27	1.00	83/341	1.13 (0.35-3.66)	
		*APOE*ε4(+)	0/6	1.00	26/59	NA	
4	Hap2: AT	All	89/321	1.00	26/112	1.07 (0.57-2.00)	0.76
	(15%)	*APOE*ε4 (-)	67/273	1.00	22/95	1.15 (0.57-2.30)	
		*APOE*ε4(+)	22/48	1.00	4/17	0.82 (0.18-3.79)	
3	Hap3: GT	All	91/356	1.00	24/77	0.89 (0.43-1.85)	0.74
	(9%)	*APOE*ε4 (-)	70/303	1.00	19/65	0.88 (0.39-1.99)	
		*APOE*ε4(+)	21/53	1.00	5/12	1.09 (0.23-5.14)	

Block2 included only one htSNP and was excluded from the haplotype analysis. Abbreviations: VaD, vascular dementia; AOR, adjusted odds ratio; CI, confidence interval; *APOE*, apolipoprotein E; NA, not applicable. All models were adjusted for age, sex, *APOE* ε4, and education years and conditional on 5-year age strata.
